# A novel lung alveolar cell model for exploring volatile biomarkers of particle-induced lung injury

**DOI:** 10.1038/s41598-020-72825-7

**Published:** 2020-09-24

**Authors:** Hsiao-Chi Chuang, Shih-Wei Tsai, Ruei-Hao Shie, Yi-Chia Lu, Sheng-Rong Song, Sheng-Hsiu Huang, Hsin-Yi Peng, Hsiao-Yu Yang

**Affiliations:** 1 School of Respiratory Therapy, Taipei Medical University, Taipei, Taiwan; 2 Institute of Environmental and Occupational Health Sciences, National Taiwan University College of Public Health, Taipei, Taiwan; 3 Green Energy and Environmental Research Laboratories, Industrial Technology Research Institute, Hsinchu, Taiwan; 4 Department of Geosciences, National Taiwan University, Taipei, Taiwan; 5 Department of Environmental and Occupational Medicine, National Taiwan University Hospital, Taipei, Taiwan

**Keywords:** Health occupations, Respiratory tract diseases, Biomarkers, Diagnostic markers

## Abstract

Quartz can increase oxidative stress, lipid peroxidation, and inflammation. The objective of this study was to explore the volatile biomarkers of quartz-induced lung injury using a lung alveolar cell model. We exposed the human alveolar A549 cell line to 0, 200, and 500 μg/mL quartz particles for 24 h and used gas chromatography–mass spectrometry to measure the volatile metabolites in the headspace air of cells. We identified ten volatile metabolites that had concentration–response relationships with particles exposure, including 1,2,4-oxadiazole, 5-(4-nitrophenyl)-3-phenyl- (CAS: 28825-12-9), 2,6-dimethyl-6-trifluoroacetoxyoctane (CAS: 61986-67-2), 3-buten-1-amine, *N*,*N*-dimethyl- (CAS: 55831-89-5), 2-propanol, 2-methyl- (CAS: 75-65-0), glycolaldehyde dimethyl acetal (CAS: 30934-97-5), propanoic acid, 2-oxo-, ethyl ester (CAS: 617-35-6), octane (CAS: 111-65-9), octane, 3,3-dimethyl- (CAS: 4110-44-5), heptane, 2,3-dimethyl- (CAS: 3074-71-3) and ethanedioic acid, bis(trimethylsilyl) ester (CAS: 18294-04-7). The volatile biomarkers are generated through the pathways of propanoate and nitrogen metabolism. The volatile biomarkers of the alkanes and methylated alkanes are related to oxidative and lipid peroxidation of the cell membrane. The lung alveolar cell model has the potential to explore the volatile biomarkers of particulate-induced lung injury.

## Introduction

Particulate matter is an important respiratory hazard in environmental and occupational health. In 2016, 91% of the world population lived in places that did not meet the WHO air quality guidelines^1^. In the United States, more than two million workers are exposed to silica-containing dust, and about 100,000 workers are involved in high-exposure tasks such as abrasive blasting, rock drilling, mining, quarrying, and construction. Chest X-ray or pulmonary function tests are not sensitive enough to detect lung injury in the preclinical stage. Early detection of lung injury caused by particulate matter is difficult, and the obstacles to screening remain unresolved^2^.

The entirety of the volatile organic compounds (VOCs) produced by an organism has been termed the volatilome^3^. Volatile biomarkers in exhaled breath might be used to assess the acute respiratory effects on subjects exposed to particles. On a physiological basis, oxidative stress plays a vital role in the pathogenesis of acute respiratory effects caused by particles^4^. When inhaling particles, the particles can induce the generation of reactive oxygen species (ROS) by direct introduction of oxidizing species into the lung, the introduction of surface absorbed chemicals that can undergo biotransformation in vivo, and the particle surface per se to elicit oxidative stress^5^. ROS causes lipid peroxidation of polyunsaturated fatty acids in the lipid bilayers of cell membranes. It produces volatile metabolites such as ethane, pentane, propane, and isopropane^6^. The volatile metabolites have been used as biomarkers to detect pneumoconiosis, a disease that is caused by the inhalation of silica particles. Yang et al. used a sensor array to analyze volatile metabolites in the exhaled breath of stone workers to screen for pneumoconiosis. The results showed that the breath profiling method had good accuracy, and the area under the receiver operating characteristic curve was 0.86 (95% CI 0.69‒1.00) for the test set^7^. Yang et al. used gas chromatography-mass spectrometry (GC–MS) to analyze the VOCs in the exhaled breath. It was found that lipid peroxidation-induced pentane and C5–C7 methylated alkanes constituted a specific fingerprint in the breath of pneumoconiosis patients. The breath test had excellent diagnostic accuracy for early-stage pneumoconiosis^8^. However, when analyzing VOCs in human breath, endogenous VOCs may be contaminated by ambient air or exogenous VOCs in the upper airways^9^. Therefore, researchers are interested in using in vitro studies to control confounding factors and explore potential volatile metabolites. In the search for new analytical technologies, downstream volatile organic compound emissions from cell cultures can be measured by GC–MS. The objective of this study was to use a lung alveolar cell model to explore potential volatile molecular biomarkers of particles-induced lung injury. We provide critical new information into the types of VOCs related to human alveolar cell injury caused by particles. To our knowledge, we are the first to measure VOCs emissions from alveolar cell cultures and to describe their VOC profiles.

## Results

**Figure 1:**
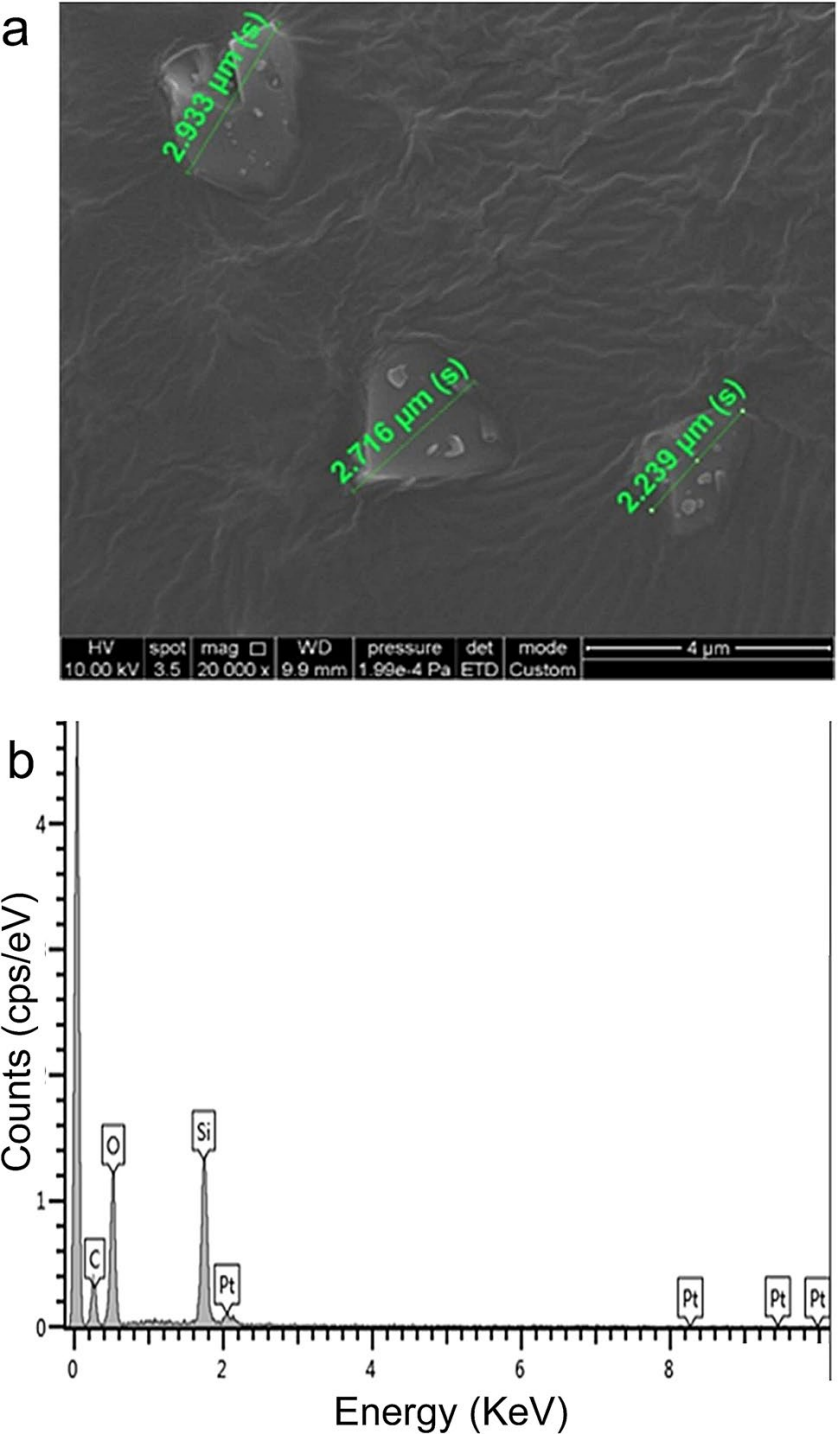
Preparation and analyses of quartz. (**a**) We examined 100 particles using scanning electron microscopy at ×20,000 magnification to confirm the aspect ratio, length, and diameter; (**b**) we analyzed the chemical composition by energy-dispersive X-ray spectroscopy. The main chemical components of the particles are Si and O.

**Figure 2:**
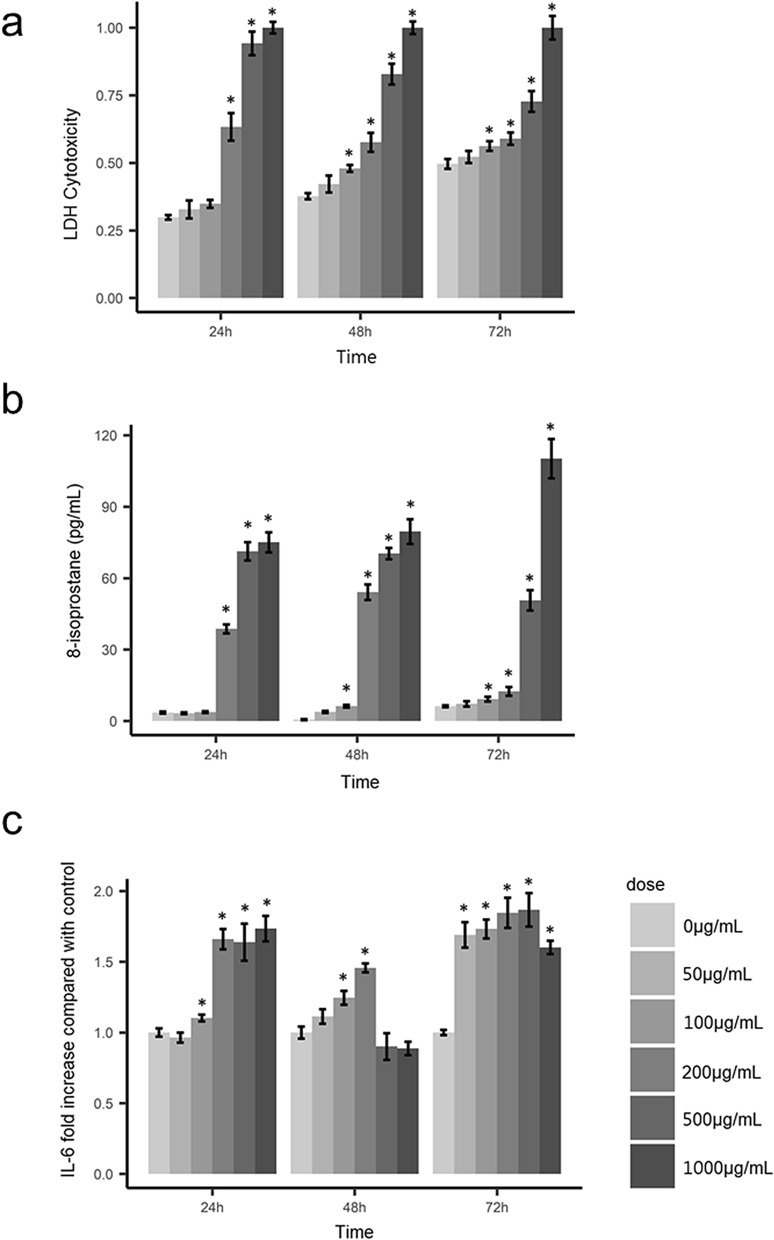
Cytotoxicity studies of (**a**) Lactate dehydrogenase (LDH) cytotoxicity (**b**) oxidative stress (8-isoprostane), and (**c**) inflammation (IL-6). Legends: The ratio is expressed as the mean ± SE of 6 replicates (*n* = 6). Mann–Whitney *U* Test *p < 0.05 vs. control.

**Figure 3:**
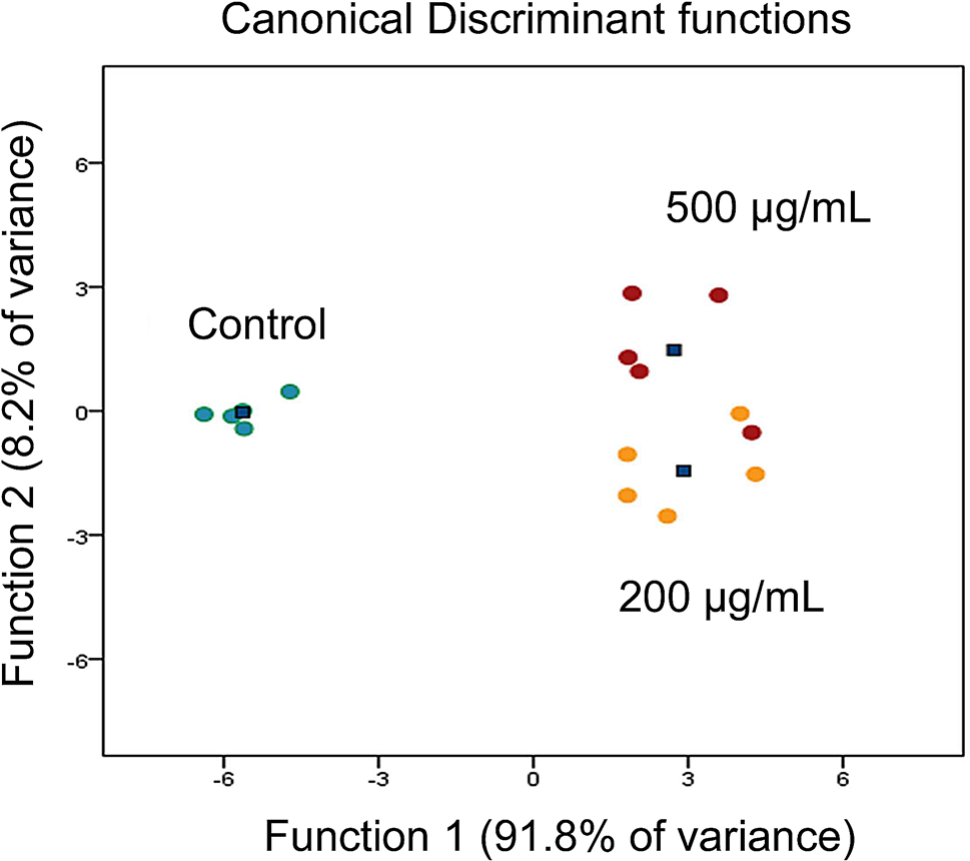
Scatter plot of canonical discrimination analysis in control, 200, 500 μg/mL. The discrimination accuracy was 93.3%. The black squares are centroid.

**Figure 4:**
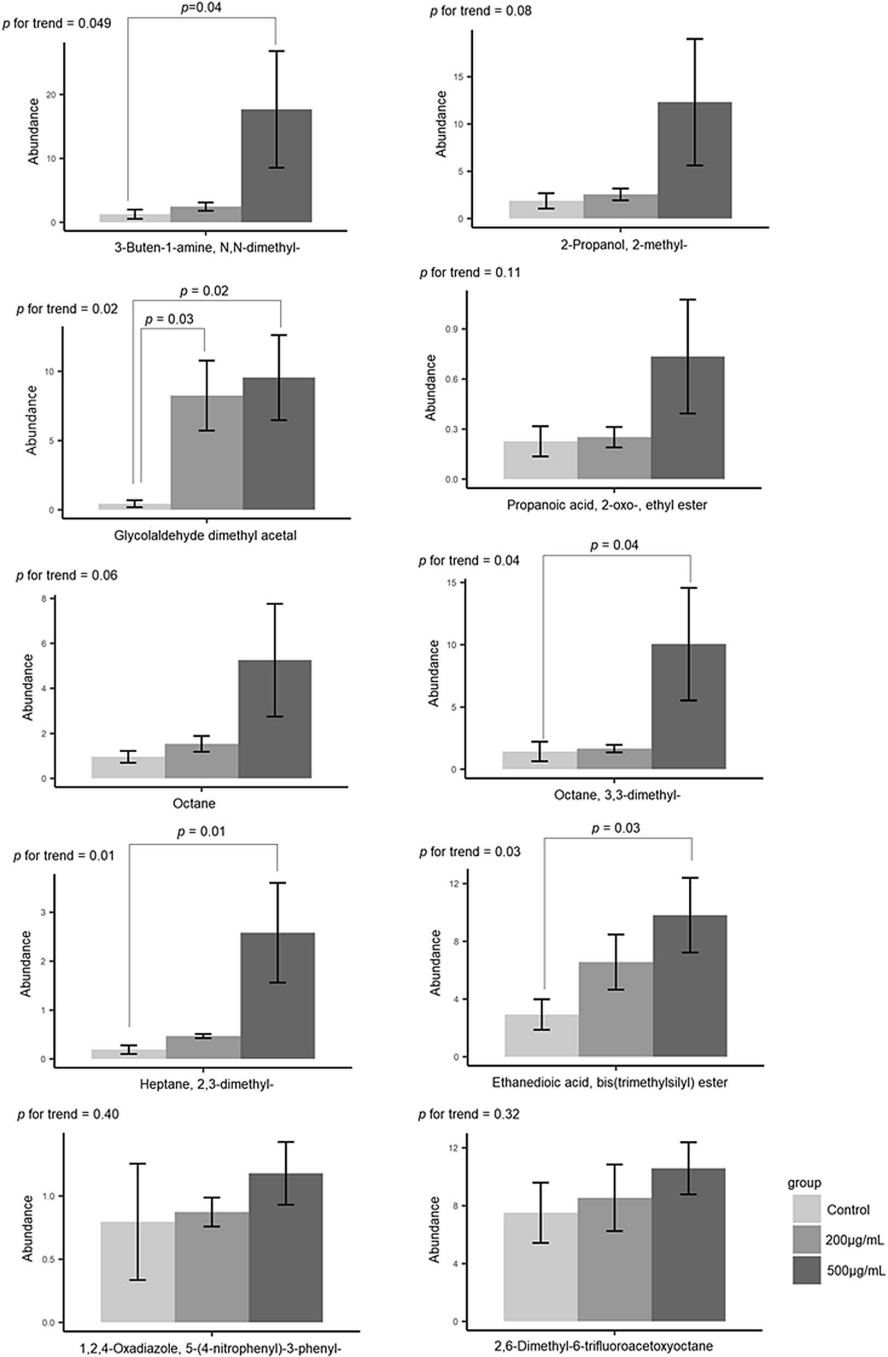
The bar chart shows the concentrations, test for trend, and pairwise comparison tests of the 10 VOCs that have concentration–response relationships with quartz exposure. Legends: The concentrations are expressed as the mean value of the abundance of the VOC and represented with a mean value ± SE. The abundance of the y-axis has been normalized by the reference feature of the external standard of bromofluorobenzene. We tested the dose–response relationships by linear trend test.

We examined 100 particles at 20,000 × magnification using scanning electron microscopy to confirm the aspect ratio, length, and diameter of particles and analyzed the chemical composition by energy-dispersive X-ray spectroscopy. We confirmed that the mean diameter of quartz particles was 2.3 µm (SD 0.8 μm), and the main chemical composition of the particles was SiO_2_ (Fig. 1). In cytotoxicity studies, we observed that when the cells were exposed for 24 h, the cytotoxic effects of cell membrane damage, oxidative stress, and inflammation showed a dose–response relationship with zero, 200, and 500 μg/mL exposure doses (Fig. 2). Based on the results of cytotoxic studies, we then exposed cells to zero, 200, and 500 μg/mL quartz particles for 24 h, and then used GC–MS to analyze the volatile metabolites in the headspace of the cells. After pre-processing the raw GC–MS spectral data with data conversion, peak detection, chromatogram building, deconvolution, normalization, alignment, and gap-filling^10^, we identified 107 compounds, which were classified into ten types of chemicals: alcohols (ten compounds), ketones (14 compounds), aldehydes (two compounds), acids/acid esters (23 compounds), amines (six compounds), organosulfur (one compound), aromatics (eight compounds), alkanes (17 compounds), alkenes (nine compounds) and other (17 compounds). The three groups of cells exposed to zero, 200, and 500 μg/mL of quartz can be discriminated well by the detected volatile metabolites (Fig. 3). Because the sample size of an in vitro study is not large, and the classification models based on the combined pool of metabolites may be over-fitting^11^. When there was a concentration–response relationship between volatile organic compounds and particle exposure, we selected it a potential volatile biomarker. We identified ten compounds that have concentration–response relationships in the headspace of the zero, 200 and 500 μg/mL quartz-exposed cells, including 1,2,4-Oxadiazole, 5-(4-nitrophenyl)-3-phenyl- (CAS: 28825-12-9), 2,6-dimethyl-6-trifluoroacetoxyoctane (CAS: 61986-67-2), 3-buten-1-amine, *N*,*N*-dimethyl- (CAS: 55831-89-5), 2-propanol, 2-methyl- (CAS: 75-65-0), glycolaldehyde dimethyl acetal (CAS: 30934-97-5), propanoic acid, 2-oxo-, ethyl ester (CAS: 617-35-6), octane (CAS: 111-65-9), octane, 3,3-dimethyl- (CAS: 4110-44-5), heptane, 2,3-dimethyl- (CAS: 3074-71-3) and ethanedioic acid, bis(trimethylsilyl) ester (CAS: 18294-04-7) (Fig. 4). Among these metabolites, five metabolites had statistical significant linear trend to the concentration of exposure, including 3-buten-1-amine, *N*,*N*-dimethyl- (CAS: 55831-89-5), glycolaldehyde dimethyl acetal (CAS: 30934-97-5), octane, 3,3-dimethyl- (CAS: 4110-44-5), heptane, 2,3-dimethyl- (CAS: 3074-71-3), ethanedioic acid, bis(trimethylsilyl) ester (CAS: 18294-04-7) (Table 1). Compared to the non-exposed cells, cells exposed to 500 µg/mL of quartz significantly increased the levels of 3-buten-1-amine, *N*,*N*-dimethyl- (*p* = 0.04), glycolaldehyde dimethyl acetal (*p* = 0.02), octane, 3,3-dimethyl- (*p* = 0.04), heptane, 2,3-dimethyl- (*p* = 0.01), and ethanedioic acid, bis(trimethylsilyl) ester (*p* = 0.03).

**Table 1:** One-way analysis of variance and trend analysis showing volatile metabolites that have concentration–response relationships with particle exposure.

Compound	CAS no.	Non-exposed control	200 μg/mL	500 μg/mL	*p* for trend
		Mean (SE)	Mean (SE)	Mean (SE)	
**Principal component 1**					
1,2,4-Oxadiazole, 5-(4-nitrophenyl)-3-phenyl-	28825-12-9	0.80 (0.41)	0.87 (0.10)	1.18 (0.22)	0.40
2,6-Dimethyl-6-trifluoroacetoxyoctane	61986-67-2	7.51 (1.86)	8.55 (2.06)	10.58 (1.61)	0.32
**Principal component 2**					
3-Buten-1-amine, *N*,*N*-dimethyl-	55831-89-5	1.27 (0.72)	2.45 (0.65)	17.69 (9.13)	0.049*
2-Propanol, 2-methyl-	75-65-0	1.87 (0.81)	2.56 (0.62)	12.31 (6.69)	0.08
Glycolaldehyde dimethyl acetal	30934-97-5	0.43 (0.25)	8.25 (2.53)	9.55 (3.07)	0.02*
Propanoic acid, 2-oxo-, ethyl ester	617-35-6	0.23 (0.09)	0.25 (0.06)	0.73 (0.34)	0.11
Octane	111-65-9	0.96 (0.26)	1.54 (0.35)	5.26 (2.50)	0.06
Octane, 3,3-dimethyl-	4110-44-5	1.43 (0.79)	1.66 (0.30)	10.05 (4.52)	0.04*
Heptane, 2,3-dimethyl-	3074-71-3	0.19 (0.09)	0.47 (0.04)	2.58 (1.02)	0.01*
Ethanedioic acid, bis(trimethylsilyl) ester	18294-04-7	2.93 (1.06)	6.56 (1.91)	9.81 (2.59)	0.03*

*p-value < 0.05.

## Discussion

This study has established a novel lung alveolar cell model to explore potential volatile biomarkers of pneumoconiosis. We exposed quartz particles to human lung cells to induce cell damage, oxidative stress, and inflammatory reactions and identified the VOCs that had concentration–responsive relationships. To the best of our knowledge, this is the first lung alveolar cell model to explore the volatile biomarkers for particulate-induced volatile biomarkers. Silica exposure affects the propanoate and nitrogen metabolisms in alveolar cells. The identified volatile biomarkers may be used to develop new breath tests for particle-induced lung injury.

Oxidative stress plays an essential role in particle-induced lung injury. When particles are inhaled and reach the alveolar, the particles are phagocytosed by alveolar macrophages. In a physiological basis, lipid bilayers are fundamental components of cell membranes. Particle induces lipid peroxidation and generates reactive oxygen species (ROS) and reactive nitrogen species (RNS)^12^. Lipid peroxidation, the oxidative degradation of membrane lipids by ROS, generates a large variety of breakdown products such as alkanes, aldehydes, ketones, alcohols, and furans^13^. Alkanes and methylated alkanes are associated with oxidative stress and lipid peroxidation^14–16^. We applied principal component analysis to identify alkanes and methylated alkanes in the major components of volatile metabolites. In the first principal component, we identified the heptane, 2,4-dimethyl- (VOC123). In the second principal component, we identified octane, 3,3-dimethyl- (VOC154), octane (VOC122), and heptane, 2,3-dimethyl- (VOC177) (Supplementary Fig. 1). However, oxidative stress is not specific to particle-induced lung injuries but also observed in some chronic diseases. For example, the heptane, 2,4-dimethyl- (VOC123) also has been reported to be released from lung cancer cell lines^17^ and acute mononuclear leukemia cell line SHI-1^18^.

This study has established a new lung alveolar cell model to explore volatile biomarkers. VOCs found in cell lines are advantageous for understanding the mechanism of VOC generation in patients. Additionally, using an in vitro study can prevent interference from exogenous contaminations, such as ambient air, food, and smoking^19^. The current method was suggested by Chen et al. to discover volatile metabolites of lung cancer that have complex mechanisms in humans^20^, and several studies have investigated the release of VOCs from human cancer cells in vitro^21,22^. In our study, we further improved the lung alveolar cell model by integrating cytotoxic tests, a closed-loop operation system, and standardized data pre-processing procedures. We have provided all the details for the lung alveolar cell model for further researchers to reproduce and validate the model.

Metabolomics is a new field of science that examines the interaction between an organism's genome and its environment that reflects the ultimate response of an organism to environmental influences^23^. Breathomics is a specialized field of metabolomics that focuses on these small molecular metabolites that can be detected in exhaled breath^24^. Exhaled breath is composed of a complex mixture of VOCs as well as vapors and aerosols derived from cells or external exposure^25^. The exhaled air carries volatile metabolites from the human body that serve as substrates and products in metabolic pathways^26^ and could be used to develop breath tests for diseases. Breath metabolite analysis might be particularly useful in lung diseases because the metabolites produced from the pathologic changes in the alveoli and epithelium of the respiratory tract are directly released into the exhaled breath^27^. Because VOC concentrations may be affected by diet, flow rate, and anatomical dead space^28^, we developed this lung alveolar cell model to establish a platform to explore the potential volatile biomarkers before expanding to human study. Current metabolomic databases usually lack the volatile metabolites. For example, the 2,6-Dimethyl-6-trifluoroacetoxyoctane was identified in a Chinese herbal product^29^. However, we searched the metabolomics databases, including Human Metabolome Database (HMDB) (https://hmdb.ca/), Kyoto Encyclopedia of Genes and Genomes (KEGG) database (https://www.genome.jp/kegg/kegg1.html)^30^, Metabolomics Workbench (https://www.metabolomicsworkbench.org/), EPA VOLATILOME: Human Breath (https://comptox.epa.gov/dashboard/chemical_lists/VOLATILOME), there was no information of 2,6-Dimethyl-6-trifluoroacetoxyoctane. We considered that 2,6-Dimethyl-6-trifluoroacetoxyoctane may not be a real biomarker and must be interpreted with caution.

Since the aim of this study was to establish a lung alveolar cell model for exploring unknown metabolites, we applied untargeted GC–MS analysis that does not use standard solutions but used a full scan method to explore unknown volatile organic compounds^31^. If there are no standard solutions, it may be difficult to distinguish the chromatographic peaks from the noise that may overlap with matrix peaks^32^. Standardized protocols are then essential to assure the reproducibility of results^33^. In this study, we used standardized protocols to process data, including retention time correction, spectral deconvolution, peak alignment, and normalization. We applied standardized procedures using the MZmine software and the NIST library to explore possible biomarkers. We used a deconvolution algorithm to extract compound peaks. The peak detection method can reduce false positives by avoiding the detection of noise peaks^10^. We repeated the entire process several times to test the reliability of data processing methods and noted that some chemicals could not be re-identified with the same parameters (3/192). Koh et al. evaluated three commonly used software for identifying compounds in metabolomics studies. They also found that although the retention time (RT) alignment was used, there were still RT alignment errors and suggested a manual check on RT alignment^34^. We encourage that further studies can validate these biomarkers with standard solutions. We suggest that further research can use other techniques, such as the two-dimensional gas chromatography-mass spectrometry (GC × GC–MS), to increase the specificity^35^.

It is essential to prevent false discovery of biomarkers from contamination. This study established an airtight sealed glove box, and we performed all procedures in this box. We checked the VOCs of GC column, SPME fiber, background air in the glove box, and empty glass flask and confirmed that there was no contamination. In our pilot study, we used disposable polystyrene culture flasks. We noted that there were high concentrations of ethenylbenzene, styrene, and ethanol in an empty flask that will cause the false discovery of volatile biomarkers of injured cell lines. Then, we replaced polystyrene culture flasks with glass flasks during the sampling of cell breath and used the glass dishes to avoid the polystyrene influence on cell growth. Disposable polystyrene culture flasks are widely used for cell culture because their design allows for space-saving stacking, and the inner surface properties are adjusted to enable the ideal adhesion of cells^36^. These polymers may emit volatile compounds, such as monomers, additives, and byproducts, which can affect the results. Schallschmidt et al*.* analyzed VOCs in new culture flasks and identified some alkanes and aromatic compounds that can be identified both in an empty flask and in flasks with cell cultures. We suggest further study should prevent the use of disposable polystyrene culture flasks in the cell breath study.

## Strengths and limitations

The advantages of this study are to conduct a cytotoxic study to confirm the effects of quartz exposure at first and then identify volatile metabolites with concentration–response relationships. This study carefully prevented contamination of ambient air, culture flasks, and instruments and used standardized methods to process metabolomics data to avoid false discovery. All research procedures were conducted in a closed-loop system. The study still has limitations. First, quartz-induced lung injury in patients with pneumoconiosis may not only involve direct cytotoxic effects on cell membranes, but also on the production of cytokines, chemokines, lipid mediators, and growth factors that induce aggregation of macrophages and fibroblasts^37^. In this study, we used only the A549 cell line and did not include macrophages or fibroblasts. The identified volatile biomarkers might not be the same as the volatile biomarkers identified in human studies that involve more complex pathological pathways. We suggest that further studies may include macrophages and fibroblasts. Second, this study used a tumorigenic lung epithelial A549 cell line. The metabolic reprogramming takes place in cancer cells and might affect the lung cell metabolites. We suggest that further study could compare the results in non-tumorigenic cell lines. Third, this study applied an untargeted metabolomic analysis that aims at exploring unknown metabolites. The identification of metabolites is based on physicochemical properties and spectral similarity with public/commercial spectral libraries^38^. However, oxidative stress is associated with many pathologies and is not specific to particle-induced responses. The volatile metabolites associated with oxidative stress identified in this study may also be common biomarkers for many diseases. The high doses of silica used in this in vitro study may be different from the human exposure dose. For further application in clinical practice, the identified metabolites must be validated in human studies and use standard compounds to compare the mass spectral profiles. Fouth, due to the column and SPME choices, our GCMS method may not have found all the chemicals.

## Conclusions

In this study, we established a novel lung alveolar cell model to explore potential volatile biomarkers for quartz-induced lung injury. We exposed human alveolar cells to quartz to induce cell injury and then analyzed the generated volatile metabolites. This study applied breathomics methods to distinguish VOCs produced by damaged and unexposed cells. The VOCs with a concentration–response relationship to quartz exposure were selected as potential volatile biomarkers to detect lung injuries caused by quartz. Alkanes and methylated alkanes are associated with oxidative and lipid peroxidation and could be used as volatile biomarkers for quartz-induced lung injury.

## Methods

We conducted a cytotoxic study to determine the optimal quartz exposure dose and time at first. We exposed quartz particles to human alveolar cells to induce cell injury and measured the levels of oxidative stress and lipid peroxidation. After confirming the cell injury through clear concentration–response relationships in the cytotoxicity tests, we determined the exposure dose and time to measure the VOCs in the headspace air of cells. The VOCs that had a concentration–response relationship with the concentration of the exposed quartz were considered as potential volatile biomarkers.

### Preparation of particles

The lung alveolar cell model has the potential to explore volatile biomarkers for particle-induced lung injury. Since atmospheric particulate matter may contain varying chemical composition, which may affect the identified volatile organic compounds, we used the quartz particle to build the model from the beginning. Particle size may affect the deposition and cytotoxic effects in the airways^39^. According to a review of aerosol deposition in human health, a particle size ranging from 2 to 6 μm affects the central and small airway^40^. We used a standard quartz sample (Mei-Ling Company, New Taipei City, Taiwan) and applied gravitational sedimentation methods to select a particle size of 2 μm for cytotoxic studies^41^. We suspended 10 g of quartz powder in distilled water. We used a D150 ultrasonic cleaner (Delta, New Taipei City, Taiwan) to perform ultrasonic stirring at a frequency of 43 k Hz for 20 min, and then allowed the mixture to settle for 7 h. We obtained particles at a depth of 10 cm below the surface of the water to obtain the particles. After confirming the physical and chemical properties, we used these particles to make stock solutions. The particles were centrifuged at 3000 rpm and 25 °C for 25 min to remove the supernatant. Then, we suspended the particles in the culture medium to prepare a 1 mg/mL stock solution. We suspended the stock solution by ultrasonic agitation for 30 min to ensure the uniform suspension before dilution with cell culture medium. We diluted the quartz particles into five doses in medium without fetal bovine serum, which was 50, 100, 200, 500, and 1000 μg/mL, respectively.

### Cell culture and treatment

To prevent VOC contamination, we used glass culture dishes instead of polystyrene dishes to prevent contamination. We obtained human alveolar epithelial A549 cells from the American Type Culture Collection (ATCC, Gaithersburg, US). Alveolar A549 cells were cultured in Roswell Park Memorial Institute media (RPMI-1640) (Merck KGaA, Darmstadt, Germany) containing 10% fetal bovine serum, penicillin, and streptomycin. We incubated the A549 cells at 37 °C and 5% CO_2_ following a standardized protocol^42^. A549 cells were seeded onto sterile 96-well plates at a density of 10^4^ cells/mL for cell viability, cytotoxicity, oxidative stress, and inflammatory tests. We exposed 0, 50, 100, 200, 500, and 1000 μg/mL quartz particles to the A549 cell lines for 24, 48, and 72 h. For each dose, we used six replicate samples.

### Determination of cell membrane damage

Lactate dehydrogenase (LDH) is a stable cytoplasmic enzyme released into the cell culture medium through the damaged plasma membrane^43^. The LDH assay determines cell membrane damage by measuring the LDH activity released by damaged cells. We used the LDH assay kit-WST kit (Dojindo Molecular Technologies, Rockville, US) according to the manufacturer's instructions. We measured the absorbance of six replicate samples for each dose and obtained a mean value.

### Determination of oxidative stress

8-Isoprostane is a biomarker of oxidative stress and has been used to measure the oxidative stress in adolescents exposed to air pollutants^44^. We measured the levels 8-isoprostane with a competitive enzyme-linked immunosorbent assay (ELISA) kit (Cayman Chemical, Ann Arbor, MI, USA) following the manufacturer's instructions.

### Determination of inflammation

Interleukin-6 (IL-6) is a pro-inflammatory cytokine that has been used to measure the inflammatory response in cultured human cells exposed to particulate matter^45^. We measured the IL-6 levels with an ELISA kit (Human IL-6 ELISA Ready-SET-Go; eBioscience, San Diego, CA) according to the manufacturer's protocol.

### Background VOCs measurements

We conducted this in vitro study in a specially designed glove box to prevent contamination from external environmental pollutants during the operation. We connected the inlet of the glove box to a high-purity nitrogen cylinder (99.995%), charcoal, and a high-efficiency particulate air filter (PALL, New York, USA). An air purifier (Honeywell HRF-V4D1, Charlotte, NC, USA) was installed in the glove box to filter residual VOCs. All flasks were flushed with neutral cleaner (Decon Laboratories Limited, Hove, UK) and then rinsed with distilled water according to standardized procedures. Before and during the experiment, we constantly supplied pure nitrogen to the glove box to maintain positive pressure. We measured background VOCs in the glove box, glass flasks, GC–MS column, and solid-phase microextraction (SPME) fiber. We extracted the headspace air of the glass flask and the glove box for 30 min following our standardized protocol. We had method blank samples in each batch of analysis to confirm that the analytical system is free of analyte contamination or interfering substances. We measured the VOCs in empty flasks, culture medium-filled flasks, GC columns, and SPME fiber coatings.

### Determination of volatile metabolites by GC–MS

The study applied an untargeted analysis and used the National Institute of Standards and Technology (NIST) Library to search for mass spectra and retention indices. Because the concentrations of volatile metabolites released from alveolar cells may be low, we applied the solid-phase microextraction technique that can measure the concentrations of VOCs in the ppb level. We seeded the A549 cells at a concentration of 9.4 × 10^5^ cells per mL in a glass culture flask with a surface area of 28 cm^2^ (each flask had 4.7 × 10^6^ cells). We exposed cells to 0 (non-exposed control), 200, and 500 μg/mL quartz particles for 24 h. For each dose, we had five flasks and five independent assays. We replaced the headspace air of the flask with synthetic air containing 90% N_2_, 5% O_2_, and 5% CO_2_, which is a suitable hypoxic culture condition for growing cancer cell lines^46^. After incubation in a humidified atmosphere (37 °C, 5% CO_2_) for 24 h, we inserted the divinylbenzene/carboxen/polydimethylsiloxane (DVB/CAR/PDMS) fiber into the flask to extract the VOCs in the headspace of the cells for 30 min. Then, the fiber was thermally desorbed in the injection port of the GC for 10 min. We performed the VOC analysis using a 6890 GC equipped with a 5973 MS (Agilent Technologies, Santa Clara, CA, USA). The column used was a DB-5MS (60 m × 0.32 mm i.d., 0.25 um film thickness) fused-dimethylpolysiloxane capillary column. The column oven temperature was programmed as follows: started at 90 ℃, held for 9 min, then increased by 5 ℃/min up to 130 ℃, and 60 ℃/min to 240 ℃. Before analyzing all samples and blanks, we performed an instrument performance check using the external standard of 4-bromofluorobenzene (BFB). For each sampling batch, we had one laboratory method blank to ensure that there was no contamination in the flask (Supplementary Figure S1).

### Pre-processing of GC–MS data

In a breathomics study, proper pre-processing of the raw data is crucial to obtain reliable data on which the statistical analysis can be performed^33^. We used MZmine 2 software to pre-process GC–MS spectral data that included data conversion, peak detection, chromatogram building, deconvolution, normalization, alignment, and gap-filling^10^. We modified some built-in parameter settings based on our pilot study ^47^ and provided all the parameters in the Supplementary Table S1. The detailed procedures for the pre-processing of GC–MS spectral data in the Supplementary information.

### Statistical analysis

First, we applied canonical discriminant analysis to assess whether the 107 volatile metabolites can be used to distinguish between control cell line, cell lines exposed to 200 μg/mL, and cell lines exposed to 500 μg/mL of particles. Then, we performed the principal component analysis with Varimax rotation and Kaiser normalization to explore the pattern of volatile metabolites from A549 cells. We identified potential volatile biomarkers for particle-induced lung cell injury by selecting the VOCs that had a concentration–response relationship between the particle exposure dose (0, 200, and 500 μg/mL) and its abundance (signal intensity). We applied the linear trend test of ANOVA and conducted the post hoc test by the Dunnett t-tests. A two-tailed *p*-value of less than 0.05 was considered statistically significant.

## Supplementary information

Supplementary Information.

## Data Availability

All the experimental procedures are publicly available in *Protocols.io* (https://protocols.io/view/cell-breath-study-y26fyhe).
