# Supplementary material for: A novel lung alveolar cell model for exploring volatile biomarkers of particle-induced lung injury
Source: Sci Rep. 2020 Sep 24;10:15700. doi: 10.1038/s41598-020-72825-7 (PMC7515894; doi:10.1038/s41598-020-72825-7)
Supplement: Supplementary file 1 — Supplementary Information. [file 41598_2020_72825_MOESM1_ESM.pdf]

Title: A novel lung alveolar cell model for exploring volatile biomarkers of particle-induced lung injury

Authors: Hsiao-Chi Chuang,[a] Shih-Wei Tsai,[b] Ruei-Hao Shie,[c] Yi-Chia Lu,[d] Sheng-Rong Song,[d] Sheng-Hsiu Huang,[b] Hsin-Yi Peng,[b] Hsiao-Yu Yang[b, e]*

[a] School of Respiratory Therapy, Taipei Medical University, Taipei, Taiwan

[b] Institute of Environmental and Occupational Health Sciences, National Taiwan University College of Public Health, Taipei, Taiwan

[c] Green Energy & Environmental Research Laboratories, Industrial Technology Research Institute, Hsinchu, Taiwan

[d] Department of Geosciences, National Taiwan University, Taipei, Taiwan

[e] Department of Environmental and Occupational Medicine, National Taiwan University Hospital, Taipei, Taiwan

* Correspondence: hyang@ntu.edu.tw; No. 17 Xuzhou Road, Taipei, Taiwan 10055. Tel.: +886-233668102

Supplementary Method

Pre-processing of GC-MS data

First, we converted the raw chromatographic data into ANDI files (analytical data interchange protocol, .cdf). The chromatographic data of the total ion chromatogram (TIC) were converted into centroid chromatographic data using the centroid peak detector tool. The chromatogram building procedure then generated an extracted ion chromatogram (EIC) for each detected mass using a minimal threshold timespan of 0.05 minutes, a noise level of 5, and a minimum peak height of 50. We used the "baseline cut-off method" algorithm for the

deconvolution step to separate overlapping peaks that appeared at the same retention time. We used the "retention time normalizer" algorithm in MZmine to prevent the influence of retention time drifts between samples. The retention time of the same ion may differ among the different samples. To match the same ion in different samples, we used the "join aligner method" in MZmine for alignment using a match score calculated based on the m/z tolerance value of 0.5 and a retention time tolerance of 1%. To address the missing values in the peak list, we applied gap filing to replace the missing value using the "same m/z and RT range gap filler" algorithm in the gap-filling procedure. After all the procedures, we matched the ions to those in the NIST Mass Spectral Search Program (version 2.0, delivered with the NIST spectra library) to identify the compounds and exported the data for further analysis. The minimum values of the match factor and the reverse match factor were both set as 700. We exported the data sets into a peak table and sorted the data by retention time (from small to large) and the average peak area value of each compound (from large to small). We kept only one ion with the highest average peak area value at a given retention time as the abundance of each compound [1]. We reprocessed the peak table following the procedure of Niu et al. [2], which included: (1) Removal of unreliable values: a variable was kept if the variable has a nonzero value for at least three out of four replicates in each group. (2) Treatment of zeros: remaining zeros after the removal of unreliable values were replaced by the minimum value in the dataset, divided by 2. (3) Normalization: In our pilot study, we calculated the relative standard deviation (RSD) of the external standard of BFB to assess the reproducibility of the measurements. In three batches of measurements, the RSD was 71.18%, which indicated that the measured values might differ in a different analysis batch. To address this problem, we normalized all the measured values in each analysis batch. The abundance of each peak was divided by the abundance of the external standard for each analysis batch. The procedure was expressed as the following equation:

$$Normalized\ value\ H_{ij} = \frac{x_{ij}}{x_{0j}}$$

where j designates the batch number, i designates the compound, and x_{0j} is the abundance of the external standard for the j analysis batch. (4) Centering: samples were centered by subtracting the mean of the compounds for all samples. The procedure was expressed with the following equations:

$$\text{Centered value } C_{ij} = H_{ij} - \overline{H}_{ij}$$

$$\overline{H}_{ij} = \frac{1}{I}\sum_{i=1}^{I} H_{ij}$$

(5) Scaling: the data were Pareto scaled by dividing each value by the square root of the standard deviation for those compounds according to the following equation [3]:

$$\text{Scaled value } \tilde{x}_{ij} = \frac{C_{ij}}{s_i}$$

where s_i is the standard deviation of each compound.

$$s_i = \sqrt{\frac{\sum_{j=1}^{J}(C_{ij} - \bar{C}_{ij})^2}{J-1}}$$

Supplementary Table S1. MZmine parameters and optimized values for GC-MS data

Step	Parameter	Value
**1) Mass detection**		
	Mass detection,	Centroid
	Noise level (positive ionization mode)	5.0E0
	Mass detection, MS level	1
**2) Chromatogram builder**		
	Min time span (min)	0.05
	Min height	5.0E1
	m/z tolerance	0.5 m/z or 100 ppm
**3) Chromatogram deconvolution**		
	Algorithm	Baseline cut-off
	Min peak height	5.0E1
	Min peak duration	0 – 10 min
	Baseline level	5.0E0
**4)Normalization**		
	Algorithm	Retention time normalizer
	m/z tolerance	0.5
	retention time tolerance	0.1
	minimum intensity	5.0E1
**5) Alignment**		
	Algorithm	Join aligner
	m/z tolerance	0.5 m/z or 100 ppm

	Weight for m/z	1
	Retention time tolerance	1%
	Weight for RT	1
**6) Gap filler**		
	Algorithm	Same m/z and RT range gap filler
	m/z tolerance	0.5 m/z or 100 ppm
**7) NIST MS Search**		
	Ionization method	No ionization
	Retention time tolerance	1%
	Max. peak per spectrum	10
	Min. match factor	700
	Min. reverse match factor	700

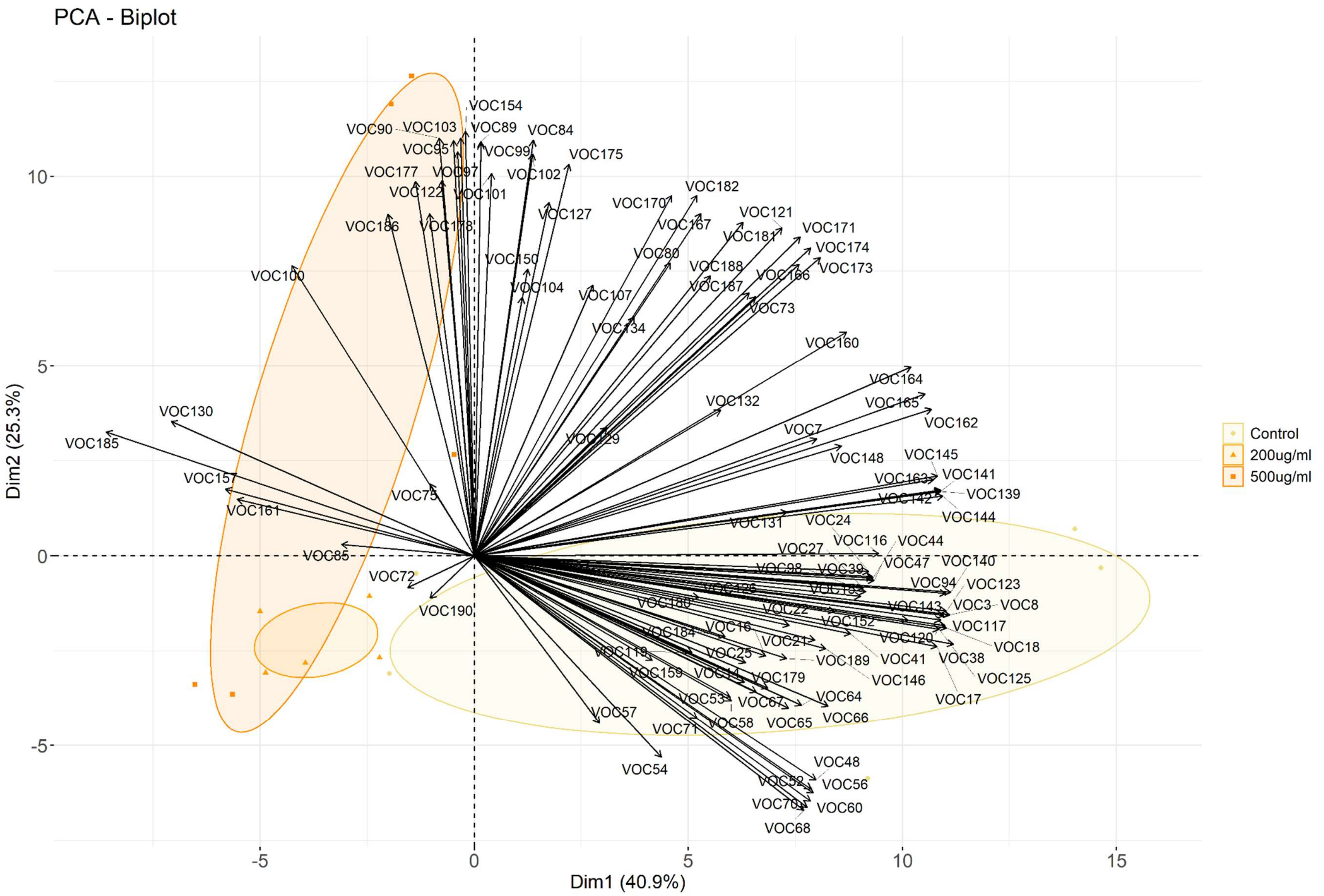

Supplementary Fig. S1. Principal component analysis of the VOCs from exposed quartz and unexposed A549 cell lines.

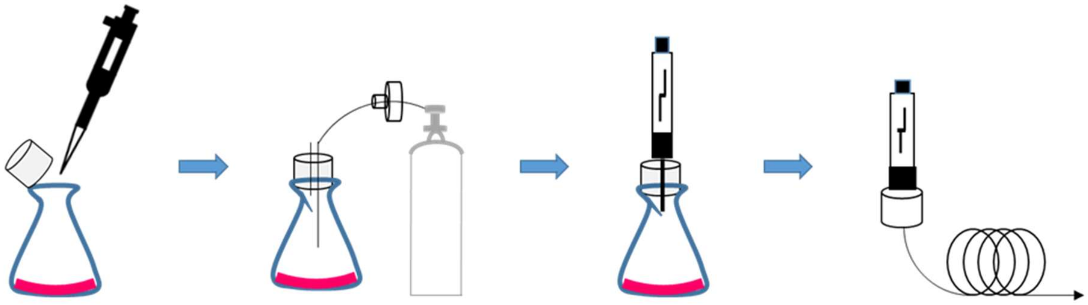

Supplementary Fig. S2. All procedures for the cell breath study were conducted in a closed-loop system to prevent contamination. After exposing the A549 cells to the quartz solution, we sealed the glass flask with a silicone septum and replaced the headspace air with synthetic air. We inserted the SPME fiber through the septum of the flask to extract the VOCs in the headspace air of the cells. VOCs extracted by SPME fibers were thermally desorbed in the injection port of the GC-MS.

References

1 Jiang, Y., Zhao, L., Yuan, M. L. & Fu, A. Identification and changes of different volatile compounds in meat of crucian carp under short-term starvation by GC-MS coupled with HS-SPME. *J Food Biochem* **41**, doi:10.1111/jfbc.12375 (2017).

2 Niu, W., Knight, E., Xia, Q. & McGarvey, B. D. Comparative evaluation of eight software programs for alignment of gas chromatography–mass spectrometry chromatograms in metabolomics experiments. *Journal of Chromatography A* **1374**, 199-206, doi:https://doi.org/10.1016/j.chroma.2014.11.005 (2014).

3 van den Berg, R. A., Hoefsloot, H. C. J., Westerhuis, J. A., Smilde, A. K. & van der Werf, M. J. Centering, scaling, and transformations: improving the biological information content of metabolomics data. *BMC genomics* **7**, 142-142, doi:10.1186/1471-2164-7-142 (2006).
